# Successful treatment of a T4 lung tumor with vertebral body invasion using fiducial markers in the thoracic spine for image-guided radiation therapy: A case report

**DOI:** 10.1186/1752-1947-5-470

**Published:** 2011-09-20

**Authors:** Anudh K Jain, John Handal, Lawrence J Solin

**Affiliations:** 1 Department of Radiation Oncology, Albert Einstein Medical Center, 5501 Old York Road, Philadelphia, PA 19141, USA; 2 Department of Orthopedic Surgery, Albert Einstein Medical Center, 5501 Old York Road, Philadelphia, PA 19141, USA

## Abstract

**Introduction:**

Paravertebral and paraspinal tumors pose a significant challenge in radiation therapy because of the radiation sensitivity of the spinal cord and the need for maximum treatment accuracy. Implantation of fiducial markers into vertebral bodies has been described as a method of increasing the accuracy of radiation treatment for single-dose stereotactic radiosurgery for spinal and paraspinal primary tumors and metastases. However, utilization of this technique has not been described for conventionally fractionated radiation therapy. This report is the first of its kind in the literature and describes successful treatment of a T4 primary lung tumor with vertebral body invasion with conventionally fractionated, image-guided radiotherapy using fiducial markers implanted in the thoracic spine.

**Case presentation:**

Our patient was a 47-year-old African-American man who presented to our hospital with a history of several months of increasing left arm pain, chest pain, dyspnea on exertion, occasional dry cough, and weight loss. He was found to have stage IIIA T4, N0, M0 lung cancer with vertebral body invasion. He had fiducial markers placed in the thoracic spine for image-guided radiation treatment set-up. The patient received 74 Gy radiation therapy with concurrent chemotherapy, and daily matching of the fiducial markers on the treatment machine allowed for treatment of the tumor while sparing the dose to the adjacent spinal cord. With one year of clinical follow-up, the patient has had regression of the tumor with only asymmetric soft-tissue thickening seen on a computed tomographic scan and grade 1 dyspnea on exertion as the only side effects of the treatment.

**Conclusion:**

Fiducial marker placement is a safe and effective technique for maximizing the accuracy and reproducibility for radiation treatment of lesions near the spinal cord. This technique may be used in conventionally fractionated radiation treatment regimens, such as those employed to treat a lung tumor with vertebral body invasion, to potentially improve clinical outcomes for patients.

## Introduction

The radiation tolerance of the spinal cord has traditionally been a dose-limiting factor in the treatment of spinal and paraspinal lesions. Intensity-modulated radiation therapy (IMRT) planning has the ability to achieve concave dose distributions that provide spinal cord sparing even if the target lesion is only millimeters away [1]. However, these treatment plans have very steep dose gradients, so a very high degree of precision is needed during radiation delivery. Even very slight set-up errors may result in significant deviation of delivery of the planned dose [2].

Fiducial marker matching is a method of image-guided radiation therapy (IGRT) used to maximize set-up accuracy. Radiopaque markers are implanted in or near the tumor site and can be imaged on the treatment machine and matched to the digitally reconstructed radiograph (DRR) from the planning computed tomography (CT) scan. The majority of published data regarding fiducial markers in the spine have pertained to methods used in stereotactic radiosurgery [3-5]. Our case report describes the application of this technique for conventionally fractionated radiation treatment of a lung tumor. The application of IGRT using fiducial markers in the spine in our patient allowed for radiation treatment of primary lung cancer with vertebral body invasion while limiting the radiation dose to the adjacent spinal cord.

## Case presentation

Our patient was a 47-year-old man who presented to our hospital with a history of several months of increasing left arm pain, chest pain, dyspnea on exertion, occasional dry cough, and a 2.3 kg weight loss over the course of the preceding three months. A physical examination showed 4 of 5 weakness of the left upper extremity but no other abnormalities.

**Figure 1:**
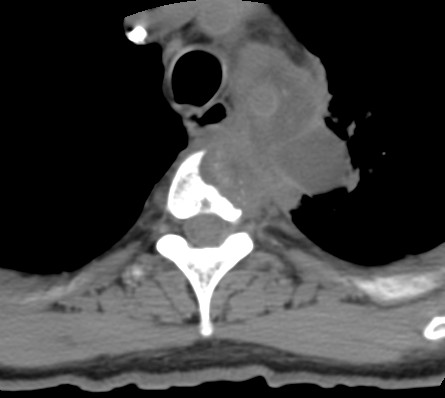
**Pre-treatment computed tomographic scan**.

He underwent CT of the chest, which showed a large left upper lobe mass measuring 6.4 cm × 3.3 cm, encasing the great vessels, and invading the T3 vertebral body (Figure 1). A video-assisted thoracoscopic biopsy of the left upper lobe mass was positive for non-small-cell carcinoma. Multiple mediastinal lymph nodes, including stations L2, L4, L5, and L7, sampled negative for tumor. A positron emission tomography (PET) scan showed hypermetabolic uptake in the mass but no mediastinal or distant metastases.

The patient was diagnosed with stage IIIA T4, N0, M0 lung cancer on the basis of the *AJCC Cancer Staging Manual*, *Seventh Edition*, staging system criteria [6]. He was offered enrollment in Radiation Therapy Oncology Group (RTOG) protocol 0617, and he consented. He was randomized to receive 74 Gy of radiation therapy using conventional daily fractions of 2 Gy with concurrent carboplatin and paclitaxel, followed by consolidation carboplatin and paclitaxel.

**Figure 2:**
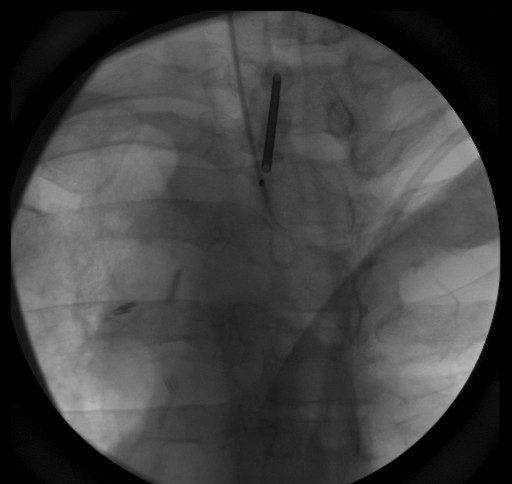
**Anteroposterior view of fiducial marker placement**.

**Figure 3:**
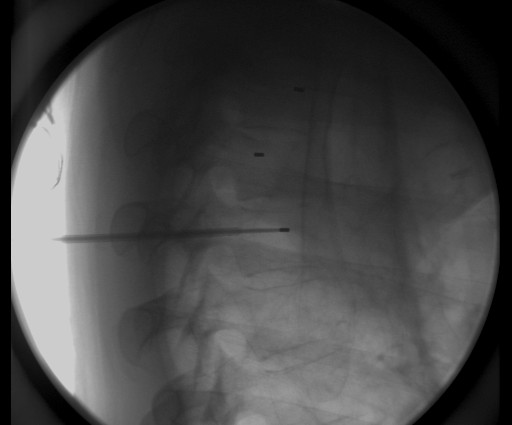
**Lateral view of fiducial marker placement**.

He was evaluated by an orthopedic surgeon (JH) for implantation of fiducial markers for IGRT because of the proximity of the tumor to the spinal cord. Markers were placed in the operating room with fluoroscopic guidance. A commercially available fiducial marker kit (CIVCO Medical Solutions, Kalona, IA, USA) was used, with 1.2 mm × 3 mm gold markers pre-loaded in 17-gauge sterile placement needles. The patient was sedated using general anesthesia. A 13-gauge Jamshidi bone marrow biopsy needle (CareFusion Corp., San Diego, CA, USA) Verified. was inserted into the pedicle of the T2 vertebral body. The pre-loaded needle containing the fiducial marker was inserted through the vertebroplasty trochar, and the gold marker was deployed into the bone (Figures 2 and 3). Bone wax was used to secure the markers in place. This procedure was repeated for the T3 and T4 vertebral bodies. The patient was then discharged to home. The only side effect he reported from the procedure was mild pain at the surgical sites, which lasted for three days and was controlled with over-the-counter pain medications.

The patient underwent CT simulation for radiation therapy planning with the use of a custom Alpha Cradle foam mold and wingboard (Smithers Medical Products, Inc., North Canton, OH, USA) Verified for immobilization. CT scans of the patient were taken during inspiration, expiration, and free breathing. CT simulation images were fused with PET images to aid in target delineation. Gross tumor volume was outlined during inspiration, expiration, and free breathing scanning and fused to form an integrated target volume. A margin of expansion of 5 mm was used to create a clinical target volume (CTV). An additional expansion of 5 mm was used to create a planning target volume (PTV). The CTV and PTV margins were decreased in the areas of bone to limit microscopic disease extension and organ motion in this area.

**Figure 4:**
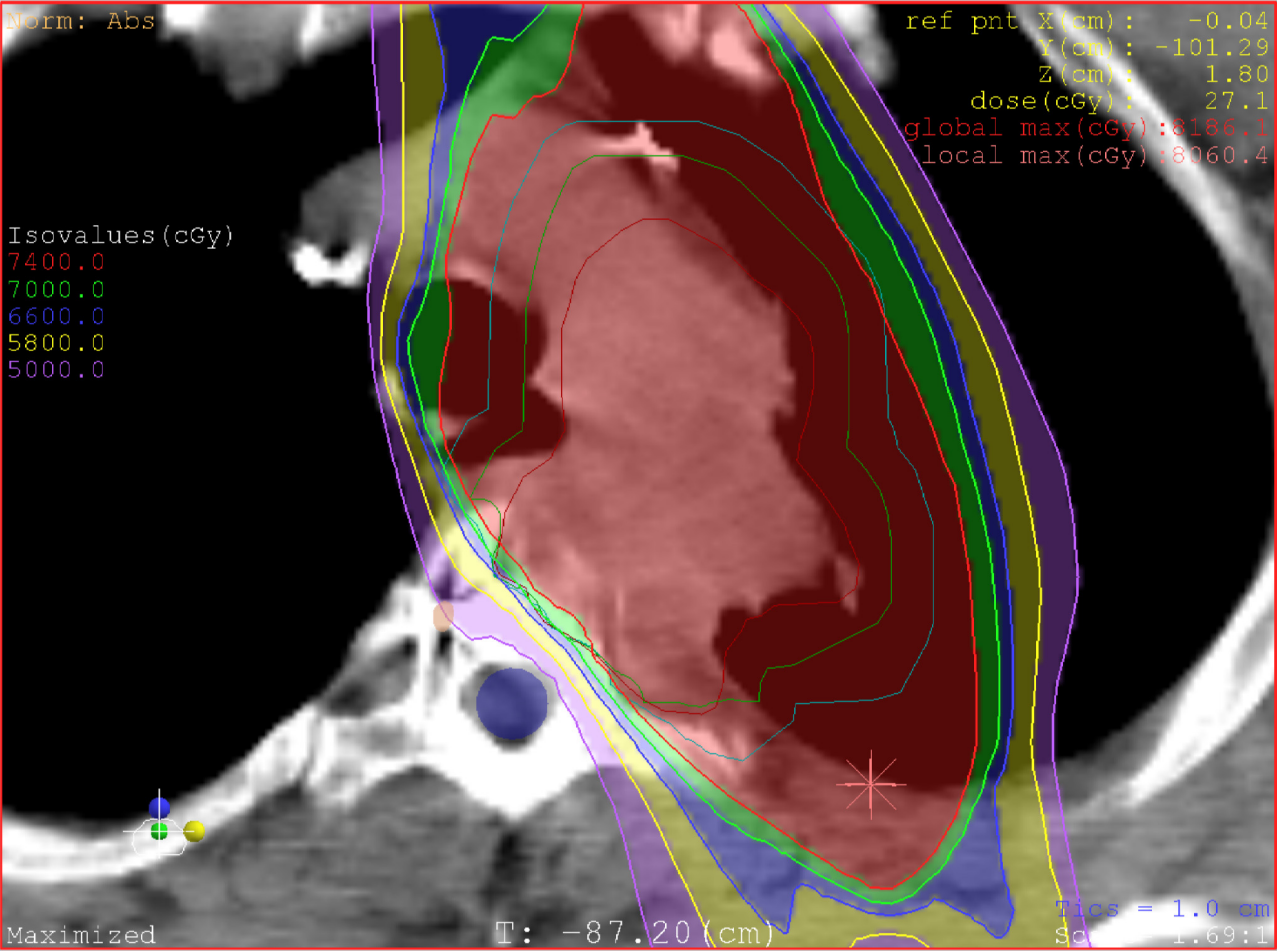
**Radiation dose distribution**.

A five-beam IMRT treatment plan was devised to provide 95% coverage of the PTV with the prescription dose of 74 Gy delivered at 2 Gy per fraction. The maximum spinal cord dose was 48.54 Gy, with a concave dose distribution and tight dose fall-off in the area of vertebral body invasion. Figure 4 demonstrates the sharp fall-off from the treatment dose of 74 Gy (red) to the approximate spinal cord tolerance dose of 50 Gy (purple). The volume of lung receiving 20 Gy was 17%. The treatment plan, including lung, heart, and esophagus dose parameters, was within the specifications of the RTOG 0617 protocol.

**Figure 5:**
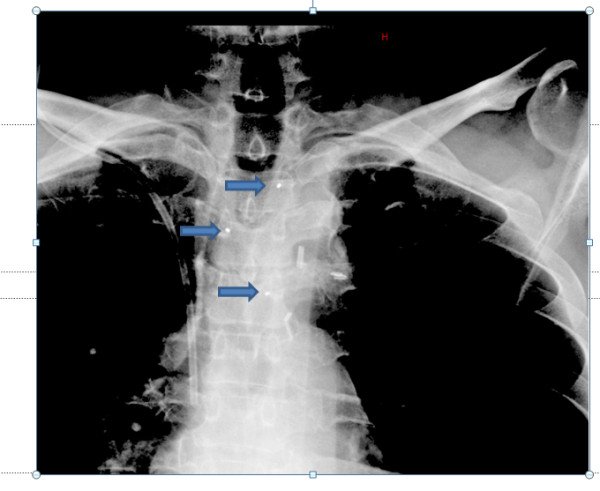
**Kilovoltage image of fiducial markers (blue arrows) (anteroposterior view)**.

**Figure 6:**
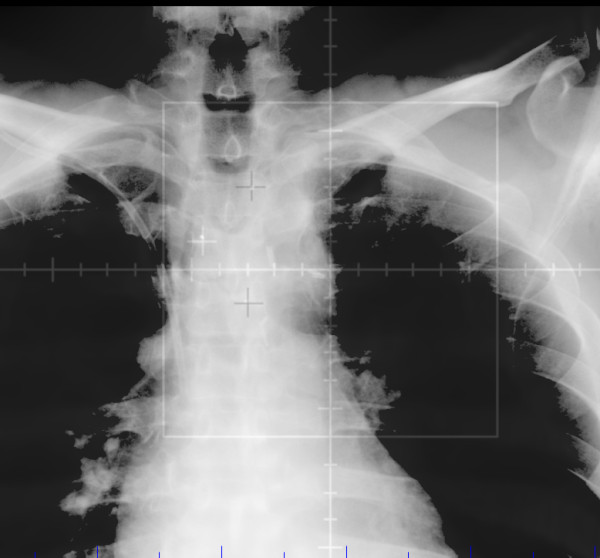
**Matching of digital reconstructed radiograph to kilovoltage image using fiducial markers (anteroposterior view)**.

**Figure 7:**
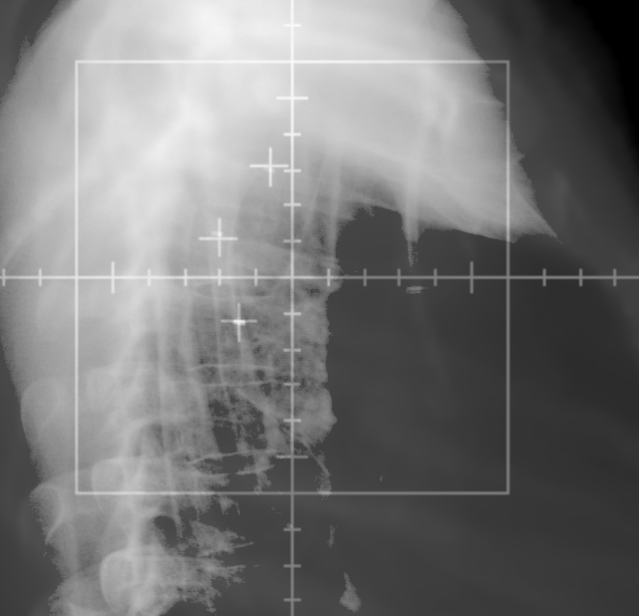
**Matching of digital reconstructed radiograph to kilovoltage image (lateral view)**.

Treatment was delivered on a Trilogy™ machine (Varian Medical Systems, Inc., Palo Alto, CA, USA) using daily kilovoltage (KV) imaging, which helped us to clearly visualize the fiducial markers (Figure 5). The position of the fiducial markers was marked on the DRR and superimposed on the KV image taken on the treatment machine, and appropriate shifts were made (Figures 6 and 7). Corrections were made in the vertical, longitudinal, and latitudinal directions as indicated. The range, median, and mean values of shifts are reported in Table 1. Migration of fiducial markers was not noted during the patient's treatment.

**Table 1:** Daily shifts at treatment set-up (in centimeters)

Direction	Range	Mean	Median
Vertical	0 to 0.7	0.2	0.2
Longitudinal	0.4 to 1.3	0.9	0.8
Latitudinal	0.3 to 1.0	0.6	0.5

The patient developed improvement in left arm pain and strength three weeks into radiation treatment. He developed grade 2 esophagitis toward the end of radiation treatment, which was controlled with diet modification and sucralfate as needed. His esophagitis resolved four weeks after treatment was completed.

**Figure 8:**
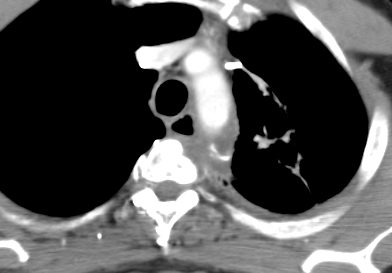
**Computed tomographic scan obtained 10 months after treatment**.

A CT scan of the chest and abdomen obtained 10 months after radiation treatment showed stable soft-tissue asymmetry in the mediastinum, with no evidence of recurrent or metastatic disease (Figure 8). At his one-year follow-up visit, the patient reported grade 1 dyspnea on exertion but was otherwise asymptomatic.

## Discussion

IGRT has emerged as a method of improving radiation treatment accuracy for tumors near the spinal cord. Three forms of IGRT for this purpose have been described in the literature: cone-beam CT (CBCT), KV X-ray imaging, and fiducial marker matching.

CBCT involves use of a CT scan that can be taken on the treatment machine. The tumor volume or normal structures on the CBCT scan are then matched with the treatment-planning CT scan, and appropriate shifts can be made. Kim *et al*. [7] used CBCT to set up single-fraction stereotactic body radiotherapy for spinal treatments and found that it provided precise target localization with accuracy within 2 mm.

KV X-ray imaging involves the use of orthogonal X-ray images that can be taken in the treatment room. These images have a higher resolution than traditionally used megavoltage (MV) images and allow for matching of patient anatomy to bony landmarks on a DRR. Yin *et al*. [8] at Henry Ford Hospital have used KV X-ray imaging with anatomy matching to vertebral bodies for intensity-modulated spinal radiosurgery and found that patient motion could be reduced to within 3 mm when coupled with good immobilization.

Fiducial markers have been used successfully in spinal radiosurgery at a number of institutions, including the University of Pittsburgh [3] and Stanford University [4]. The implantation of markers involves an invasive procedure with associated risk. Gerstzen *et al*. [5] reported a series of spinal radiosurgery procedures where fiducial tracking was used in 30 cases, and they reported one durotomy secondary to the marker implant procedure. Movement, or migration of markers, can also interfere with the use of fiducial markers for accurate treatment set-up. Shirato *et al*. [9] reported marker migration in one of three patients who underwent transcutaneous insertion of spinal markers for radiotherapy.

However, there are data suggesting that there may be increased accuracy of fiducial markers with the use of other forms of IGRT. Watchman *et al*. [10] compared KV X-ray imaging and fiducial marker matching for the set-up of 18 treatment fractions of stereotactic spinal radiotherapy. They found that matching to implanted markers was consistently more accurate, by approximately 0.5 mm, which also led to significant sparing of the spinal cord dose.

**Figure 9:**
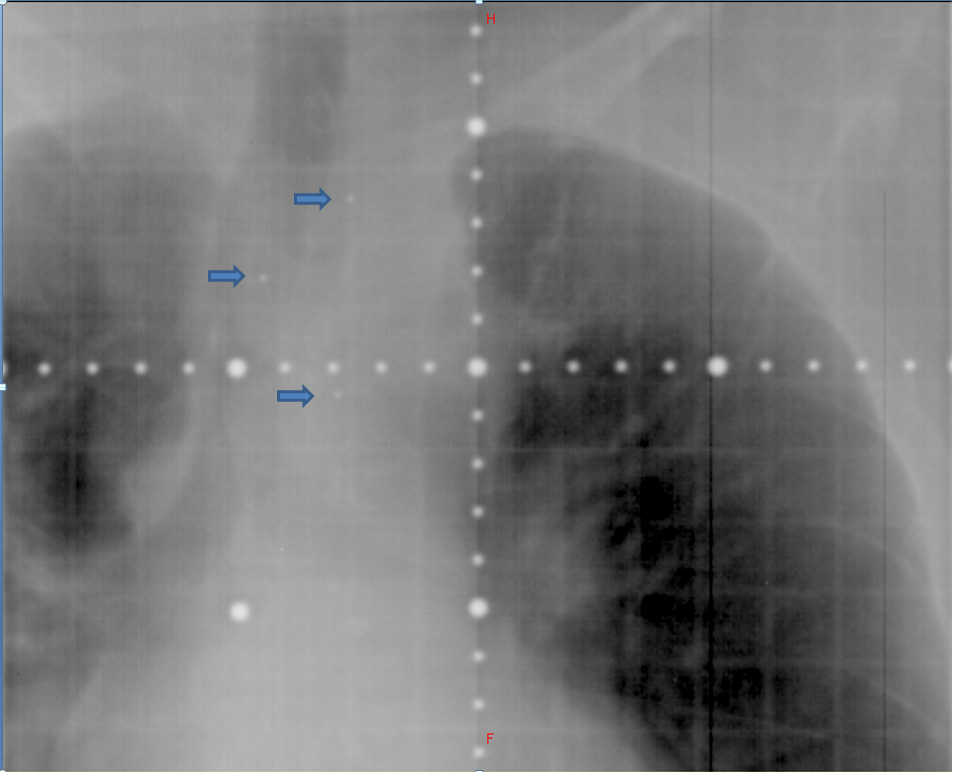
**Megavoltage image of fiducial markers (blue arrows) (anteroposterior view)**.

Fiducial markers carry several practical advantages. The markers can be clearly visualized on MV imaging (Figure 9). Some centers that may not have newer modalities such as KV imaging or CBCT available can still use MV imaging for the daily set-up to fiducial markers. The daily matching process takes only a few minutes for each patient, so it can be implemented in busy clinical departments with minimal impact on staffing needs and machine time. The use of spinal markers also limits the possibility of human error during the matching process, such as matching to the wrong vertebral body, which can occur when using other forms of IGRT.

The case of our patient shows an extension of this technique from stereotactic radiosurgery to conventionally fractionated radiation therapy. Patients who have spinal or paraspinal tumors who are not candidates for radiosurgery because of tumor factors such as large tumor size or other clinical reasons (such as indications for concurrent chemotherapy) may benefit from the use of spinal fiducial markers for conventionally fractionated radiation therapy.

## Conclusions

Fiducial marker placement is a safe and effective technique for maximizing the accuracy and reproducibility of radiation treatment to lesions near the spinal cord. The procedure can be performed on an out-patient basis and with little discomfort to the patient. This technique can be used in combination with IMRT in conventionally fractionated radiation therapy to potentially improve clinical outcomes for patients.

## Consent

Written informed consent was obtained from the patient for publication of this case report and any accompanying images. A copy of the written consent is available for review by the Editor-in-Chief of this journal.

## Competing interests

The authors declare that they have no competing interests.

## Authors' contributions

AKJ supervised all aspects of radiation treatment planning and delivery, analyzed and interpreted the patient data, and performed literature review. JH performed the implantation of the fiducial markers. LJS analyzed and interpreted the patient data and conducted the literature review. All authors read and approved the final manuscript.
